# Toxin Profile of Two *Gymnodinium catenatum* Strains from Iberian Coastal Waters

**DOI:** 10.3390/toxins14110762

**Published:** 2022-11-04

**Authors:** Joana F. Leal, Gabriel Bombo, Hugo Pereira, Bernardo Vicente, Ana Amorim, Maria L. S. Cristiano

**Affiliations:** 1 Centre of Marine Sciences (CCMAR) and Department of Chemistry and Pharmacy, Faculty of Science and Technology, University of Algarve, Campus de Gambelas, 8005-139 Faro, Portugal; 2 GreenCoLab, Green Ocean Association, Universidade do Algarve, Campus de Gambelas, 8005-139 Faro, Portugal; 3 Centro de Ciências do Mar e do Ambiente, Aquatic Research Network, Faculdade de Ciências, Universidade de Lisboa, 1749-016 Lisboa, Portugal

**Keywords:** bioprospection, toxic microalgae, *Gymnodinium catenatum*, paralytic shellfish toxins, PST bioconversion

## Abstract

*Gymnodinium catenatum* has been the main species responsible for paralytic shellfish poisoning events along the Portuguese coast (Iberian Peninsula), causing bans on bivalve harvesting that result in huge economic losses. This work presents the characterization of two novel isolates of *G. catenatum* regarding their growth and toxin profiles. Laboratory growth experiments revealed that, although low growth rates were obtained during cultivation, the cell yields were high compared to those reported in the literature. Evaluation of the toxin profiles, by HPLC-FLD, essentially confirmed the typical composition of toxins of this regional population (Iberian Peninsula), namely, the absence or low representation of the toxins dcNEO, GTX1,4 and NEO and a higher ratio of the toxins C1,2, GTX6 and GTX5. However, the percentage of the identified toxins varied among the strains of this study (under the same isolation, growth, and analysis conditions), and also differed from that of other strains described in the literature. Interestingly, we found a comparatively high abundance of dcSTX in both strains, relative to the other toxins, and an unquantifiable amount of C3,4 toxins. In addition to the geographic relationship between toxin profiles, chemical conversions among toxins may explain some differences encountered in the toxin profiles of *G. catenatum* strains.

## 1. Introduction

Dinoflagellates are a group of unicellular eukaryotes known as an important source of marine toxins [1]. Several marine toxins are harmful to human health through the ingestion of food containing these toxins (e.g., bivalves). This group of microalgae is gaining increasing relevance for different biotechnological applications, particularly in drug discovery, due to the high diversity of toxins with important biological activities [2]. In this context, bioprospection of novel strains is key to increasing the current portfolio because different isolates of the same species are known to display distinct levels and toxin profiles [3,4,5,6,7,8,9].

Among the dinoflagellate species associated with human poisoning syndromes, those producing paralytic shellfish toxins (PST) stand out because of their significant contribution to the number of harmful algae bloom (HAB) events reported worldwide (35% of the events associated with seafood toxins between 1985 and 2018) [10,11]. The effects of PST on human health are a cause for concern. Some of the typical symptoms are facial numbness, paralysis, and respiratory arrest, but in acute situations poisoning may lead to death [12,13,14,15]. According to European regulation 853/2004, the maximum regulatory limit for PST is 800 micrograms of STX (saxitoxin) equivalents per kilogram of shellfish meat. [16]. 

**Figure 1:**
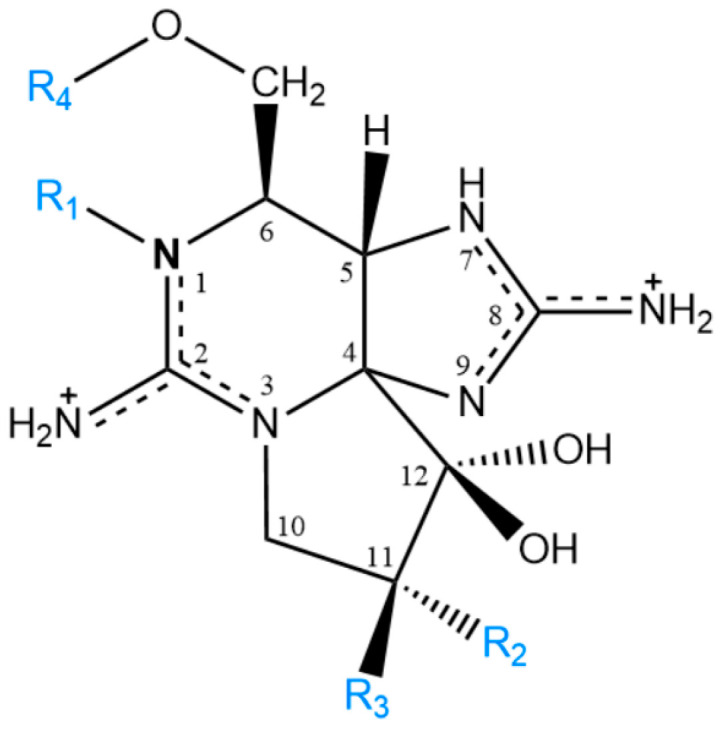
Representation of the molecular structure of PST. Substituents and subgroups: R_1_ = -H or -OH; R_2_ and R_3_ = -H or -OH or -OSO_3_^−^; R_4_ = -CONH_2_ (carbamoyl) or -CONHSO_3_^−^ (*N*-sulfocarbamoyl) or -CO(C_6_H_4_)OH (benzoyl) or -H.

PST represent a group of compounds consisting of more than 50 analogues [17]. Structurally, PST are highly polar and hydrophilic molecules comprising a 3,4-propinoperhydropurine tricyclic system and two guanidine groups. Structural diversity arises from the presence of chemically diverse substituents at different positions in the molecule that affect the toxicity of each analogue. In general, PST are classified into four main subgroups based on the substituent group in R4 (Figure 1): carbamoyl- (STX; NEO; GTX1,4; GTX2,3), *N*-sulfocarbamoyl- (GTX5; GTX6; C1,2; C3,4), benzoyl- (GC1-6) and decarbamoyl-PST (dcSTX; dcNEO; dcGTX1,4; dcGTX2,3) [18,19,20]. Regarding their relative toxicity, it is consensually accepted that the carbamoyl subgroup is the most potent followed by the decarbamoyl and *N*-sulfocarbamoyl subgroups. The toxicity potential of the benzoyl subgroup has not been clearly defined so far; yet, some studies point to a high potency [21,22].

In marine ecosystems, PST are produced by species belonging to three different dinoflagellate genera: *Gymnodinium*, *Alexandrium* and *Pyrodinium* [23]. Among them, *Gymnodinium catenatum* is a species associated with major outbreaks that lead to shellfish toxification by PST in different parts of the world [24] (and references therein). *Gymnodinium catenatum* from the Gulf of California was first identified and described in 1939 [25], and since the 1980s it has been held responsible for PST outbreaks in Portugal [5,26,27,28]. Regarding the toxin profile of *G. catenatum*, variations among strains and populations from different geographical regions have been observed [3,4,6,9], although, some toxins appear to be common to most reports with C1,2 toxins usually featuring as some of the most abundant [24].

In this context, the present work provides a characterization of two new isolates of *G. catenatum* from the Portuguese coast (NE Atlantic), regarding their growth performance and toxin profiles. The results are discussed in the context of the available knowledge concerning the biogeographical variability of the toxin profile of *G. catenatum*, mainly in the Iberian Peninsula. Reference is made to the possible evidence for interconversion relationships among toxins that may account for differences in the toxicological profiles of *G. catenatum* strains.

## 2. Results

### 2.1. Growth Characterization of G. catenatum Cultures

**Figure 2:**
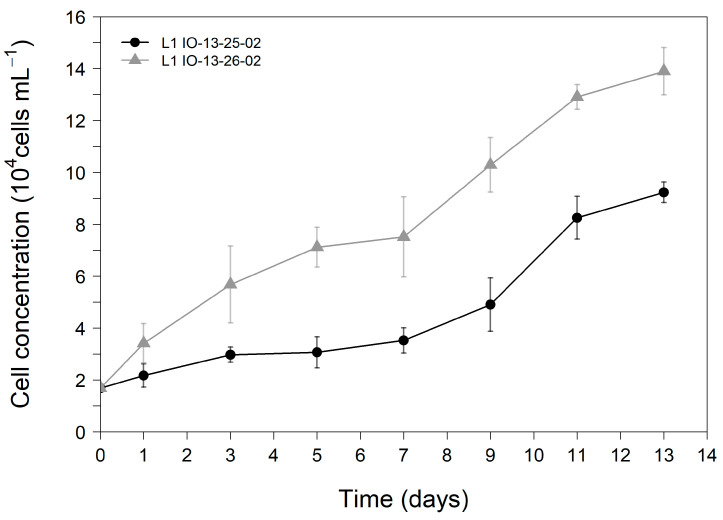
Growth curves of *G. catenatum* strains IO13-25-02 and IO13-26-02 for 13 days in L1 culture medium (n = 3).

The growth curves followed over 13 days for both strains of *G. catenatum* are shown in Figure 2. Triplicate cultures of both strains were inoculated with an initial cell concentration of approximately 1.69 × 10^4^ cells mL^−1^. The growth curves of both strains were characterised by a long lag phase until day seven followed by a short exponential phase lasting until day 11, when they entered stationary phase. The growth curves demonstrate that *G. catenatum* IO13-26-02 displayed a higher growth performance registering statistically higher cellular concentrations after the third day of cultivation (*p* < 0.05) than those recorded for *G. catenatum* IO13-25-02. Average maximum cell yields of (9.24 ± 0.39) × 10^4^ cells mL^−1^ and (13.90 ± 0.91) × 10^4^ cells mL^−1^ were registered on the final day of the trial for *G. catenatum* IO-13-25-02 and IO-13-26-02, respectively (Figure 2).

The growth rate of *G. catenatum* IO13-26-02 (0.162 ± 0.005 d^−1^) was significantly higher (*p* < 0.05) than that of *G. catenatum* IO13-25-02 (0.130 ± 0.003 d^−1^). In turn, the duplication time (number of days) was longer for IO13-25-02 (5.32 ± 0.13) than for IO13-26-02 (4.29 ± 0.13).

### 2.2. Determination of PST Profile

The PST profile was evaluated using the high-performance liquid chromatography with fluorescence detection (HPLC-FLD) method. LOQs (limits of quantification) ranged between 0.02 and 0.42 μM and all *R*^2^ (determination coefficient) ≥ 0.9990 (Table S1, in the supporting information—SI). In all sample extracts, toxins eluted between 4 and 10 min. The blank, oxidized with peroxide or periodate, did not reveal peaks in this time interval. Similarly, the unoxidized sample extracts did not reveal naturally fluorescent co-extractives corroborating that, under these analysis conditions, these toxins are only detected by a pre-oxidation reaction.

With respect to toxin profiles, analysis of both strains revealed undetectable amounts of the toxins GTX2,3, dcNEO and NEO. The toxins C3,4, GTX1,4 and STX were detected but their amounts were below the corresponding LOQs. The toxins dcGTX2,3, C1,2, dcSTX, GTX5 (or B1) and GTX6 (or B2) were quantified, revealing concentrations ranging from 0.07 to 1.36 µM. To determine the toxin concentration (femtomole, fmol) per cell, the cell concentration of each replicate and each strain was considered. Then, the average values (±standard deviation, SD) were calculated considering the biological replicates for each strain. The results are shown in Table 1. The molar fraction was estimated only based on quantifiable toxins. To calculate the molar fraction (%), the ratio between the concentration of each toxin (µM) and the sum of the concentration (µM) of all toxins, for each replica of each strain, was determined and then multiplied by 100. The toxin content differed between the strains (Table 1 and Figure S1, in the SI). The concentrations of dcGTX2,3 and C1,2 were higher in strain IO13-25-02 than in IO13-26-02. Inversely, dcSTX concentration was higher in strain IO13-26-02 than in IO13-25-02. The GTX5 content was similar in both strains. The GTX6 content was not significant in IO13-25-02; whereas, in IO13-26-02 it reached 4.2 ± 0.6 fmol cell^−1^ corresponding to the second most abundant toxin of this strain. In both strains, dcSTX was the most abundant analogue.

Concerning the toxicity levels, the average global toxicity of the IO13-26-02 strain (10.6 fmol cell^−1^) was higher than that of IO13-25-02 (8.3 fmol cell^−1^). These results were determined by the sum of the average concentrations of each quantified toxin (*i*), considering the correspondent toxicity equivalence factors (TEFs), according to Equation (1). The TEFs used are those proposed by the European Food Safety Authority (EFSA) [29] and presented in Table 1. For the epimeric pairs (dcGTX2,3 and C1,2), the TEF values presented in the table correspond to the highest value of the pair.

(1) $$Global\;toxicity=\sum toxin_{i}\times TEF_{toxin_{i}}$$

## 3. Discussion

Microalgae and their associated bioactive metabolites have great environmental relevance and enormous potential for applications both in the biotechnological and medicinal fields. Therefore, a deeper understanding of the biological and chemical processes, including the chemical composition and properties of metabolites, will translate into research developments that are better suited to the benefit of society. For instance, in the case of PST produced by *G. catenatum*, earlier studies uncovered the anaesthetic properties of some of the molecules (analogues), namely, STX and NEO [30,31,32]. In this work we observed a greater abundance of dcSTX and C1,2 or GTX6 on the strains of *G. catenatum* analysed. C1,2 and GTX6 toxins have a low toxicity potential but, according to EFSA, dcSTX has a similar toxicity potential to STX and NEO [29], which present the highest toxicity potential (1.0). Thus, although dcSTX toxicity has been revised downwards more recently [33], it remains a potential anaesthetic candidate.

Despite the high sensitivity of *G. catenatum* to environmental and cultivation conditions (e.g., light, temperature, culture container), high cell density of both strains was obtained in this work, compared to the results previously reported in literature. For example, *G. catenatum* MEL11, from the East China Sea, reached a maximum cell density of (1.14 ± 0.15) × 10^4^ cells mL^−1^ at 20 °C [34]. The high cell yields are probably related to the high starting cell concentrations used for the inoculation of cultures in the present work. On the other hand, both *G. catenatum* strains reported here displayed a low growth rate compared to *G. catenatum* MEL11, which attained 0.66 ± 0.19 d^−1^ and a duplication time of 1 day [34], and when compared to *G. catenatum* BAPAZ-10 from Mexico, which had a duplication time of 2.53 days [35]. The former work highlighted the significantly faster growth of *G. catenatum* BAPAZ-10 when compared to Iberian strains. However, *G. catenatum* LIMS-PS-2604 from the Korean coast showed a comparable growth rate of 0.21 d^−1^ [36].

Regarding the profile of toxins produced by the *G. catenatum* strains, there are many factors that may account for intra-specific differences. The life-cycle stage of the original isolation (e.g., vegetative stage vs. resting cyst), growth conditions (e.g., light, temperature, salinity, microbiome), sample preparation and analysis methods (e.g., HPLC-FLD vs. LC-MS), as well as the genetic signature are examples of factors that may influence the toxin profile [3,7,24,37,38]. In our study, two strains isolated from the same bloom, which had the same age in culture, were grown under the same abiotic culturing conditions. Care was taken so that the collection of extracts containing the toxins and sample preparation before analysis were performed simultaneously. Pre-oxidation and analysis by HPLC-FLD were performed sequentially in a few days, adopting the same conditions for both strains. Even so, significant differences were observed in the toxin profiles of the studied strains thus highlighting the existence of other possible factors that may affect the observed intra-strain variability.

**Figure 3:**
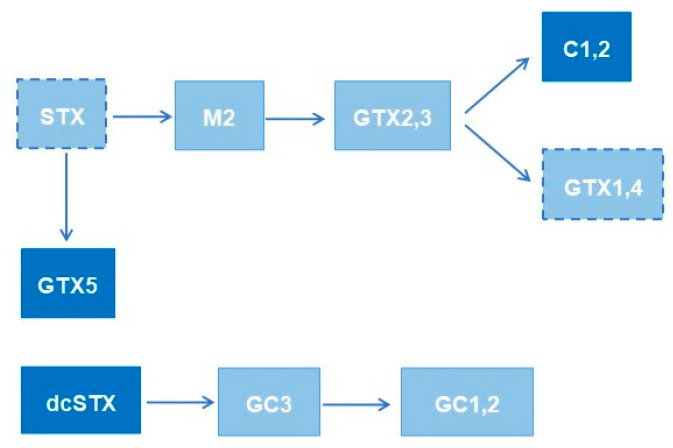
Bioconversion reactions reported in the literature for *G. catenatum.* The darker coloured boxes identify the toxins quantified in this work, while the boxes with dashed boundaries represent the toxins detected but with concentrations below the LOQ.

One aspect that should be considered is a set of bioconversions at the molecular level that may also influence the toxin profile. In fact, previous studies have already demonstrated that metabolic transformations occur in dinoflagellates, namely, in *G. catenatum*. In our previous work [20], an extensive review of the bioconversions of PST that occur in different species (dinoflagellates, bivalves, and humans) was carried out. Based on that information, Figure 3 presents a summary of the bioconversion reactions reported in the literature for *G. catenatum* [19,39,40] involving the toxins that were quantified in this study. The main reactions reported by those authors are sulfonylations (GTX2,3 to C1,2; STX to GTX5; M2 to GTX2,3). They are mediated by specific sulfotransferase (ST) enzymes (*N*—ST or *O*—ST) and use the sulfate group from 3′-phosphoadenosine 5′-phophosulfate (PAPS) as the sole source [39,40]. The activity of these enzymes is species specific, and their substrate affinity appears to be affected by the presence of some substituent groups (e.g., –OH) [20,39,41]. Additionally, oxidation (GTX2,3 to GTX1,4) and hydroxylation (STX to M2) reactions are also possible. Although the kinetics of all these reactions are not known, the different reaction rates and the competition for specific substrates could be a hypothesis to justify the different toxin profiles. This has been reported for clams (bivalves), for instance, where the bioconversion into decarbamoyl toxins (e.g., dcSTX) from *N*-sulfocarbamoyl toxins (e.g., GTX5) seems to be faster than from carbamate toxins (e.g., STX) [42,43,44]. The impossibility of quantifying, in this work, all the toxins eventually involved in these reactions does not allow us to confirm this hypothesis with certainty, but the issue should be kept in mind for future studies.

*G. catenatum* seems to have a relatively conservative genetic profile [3], but the relative abundance of each PST produced by this dinoflagellate differ between strains of different geographic origins [3,4,5,6,7,8,9,27,45]. In this work, a comparison (Table 2) of our results with the toxin profiles presented by other authors [3,5,7,8,9,27,46] was made, considering regional strains from Iberian Peninsula. The data in this table, in mol%, were normalized to allow direct comparison of ratios for the same toxins. The values show that our results are mostly within the range disclosed in the literature. Considering the strains from the Iberian Peninsula, the toxin profile seems to be characterized by the absence or low representation of the toxins dcNEO, GTX1,4 and NEO, unlike other strains from Mexico, where NEO is more representative [6]. Although with low representation, the toxins GTX2,3 (<5%) and STX (<13%) were also quantified in some samples, mostly those associated with strains from the Atlantic and Mediterranean areas collected before 2007. Additionally, the toxin profile of these regional strains seems to be characterized mainly by the presence of C-toxins (C1,2 and C3,4), GTX5, GTX6, dcSTX and dcGTX2,3 (in lower amounts). However, the relative percentage of each (pair of) toxin(s) is variable, which may be attributed to the different factors referred above. In fact, some studies have associated the variation of some physicochemical parameters with the variation of the toxins profile. For example, Band-Schmidt and co-authors (2014) observed the decrease and increase in the molar percentage of C1,2 and GTX5,6, respectively, when they increased the temperature from 16 to 33 °C [37]. However, a narrower temperature range (20–26 °C) does not seem to be enough to change the toxin profile, as suggested by other authors [34].

In the specific case of C3,4 toxins, the methods of preparation and analysis may have even more impact on their quantification. For the first time, we tried to directly quantify the C3,4 toxins, with calibration curves prepared from a CRM, but these toxins were not present in the samples in quantifiable amounts. Note that indirect quantification, by acidic hydrolysis, has been the preferred method for quantification of these toxins in AOAC Official method 2005.06 as the existence of a CRM for C3,4 is very recent. This is an issue that may eventually contribute to the differences in abundance of C3,4 in relation to other studies focused on the Iberian Peninsula, because different toxin profiles could result from differences in the sample preparation and analysis methods, as mentioned above.

Another relevant finding of this work is the higher percentage of dcSTX (average values between 40 and 50%) compared to previous studies, in which most values are below 15% (Table 2). Only two strains (IO13-17 and IO13-06) presented values closer to the values found in this work. Chemically, dcSTX may originate from the hydrolysis of STX or GTX5 (B1) at R4 substituent group (Figure 1). From the analysis of Table 2 there seems to be an inverse trend between dcSTX and GTX5 for most of the studies presented, that is, higher molar percentages of dcSTX seem to be associated with lower percentages of GTX5, and vice versa. Considering these data, it is hypothesized that part of the amount of dcSTX quantified in this work results from the molecular conversion from GTX5 by hydrolysis. Although it cannot be extrapolated, this trend appears to agree with observations previously reported for other species, namely, clams, which revealed a faster bioconversion to decarbamoyl toxins (e.g., dcSTX) from *N*-sulfocarbamoyl toxins (e.g., GTX5) than from carbamoyl toxins (e.g., STX) [42,43].

## 4. Conclusions

Among regional populations (Iberian Peninsula), there are differences in toxin profiles between strains. This study revealed that the studied strains differ from other strains from the same region by having a higher percentage of dcSTX and a lower amount of C3,4. It also reinforces the absence or low representation of the toxins dcNEO, GTX1,4 and NEO as a typical feature of regional Iberian strains. Differences in content between the two strains studied were also observed: the concentrations of dcGTX2,3 and C1,2 were higher in IO13-25-02 than in IO13-26-02, while the dcSTX concentration was higher in IO13-26-02 than in IO13-25-02. Also, in this work, some bioconversion reactions are disclosed and considered as a possible promoter of the different toxin profiles. An apparently reverse trend between dcSTX and GTX5 (molar percentage) suggests that dcSTX results from the molecular conversion from GTX5 by hydrolysis. These studies are relevant to better characterize the toxin profile of *G. catenatum* and consolidate the existence of a biogeographic profile. In addition, the combined data may allow the anticipation of the global toxicity of a strain, which may be higher or lower, depending on its toxin profile in each geographic region.

## 5. Materials and Methods

### 5.1. Sampling and Isolation Procedures

The clonal strains of *G. catenatum* used in the present study (IO13-25-02 and IO13-26-02) were obtained upon reisolation of single chains from non-clonal cultures. These were originally established from the isolation of wild cysts, putative planozygotes and motile chains from phytoplankton net samples collected in Lisbon Bay (38°41′37″ N 9°24′52.5″ W), in September 2018 during a bloom of *G. catenatum*. Once established, cultures were maintained in L1 medium [47] salinity 33 ppt, at 19.0 ± 1.0 °C, under 12 h:12 h light: dark cycle and photosynthetic photon flux density (PPFD) of ca. 40 μmol photons m^−2^s^−1^. Cultures were maintained in the algae culture collection at Lisbon University (ALISU).

### 5.2. Culture Growth Conditions

Cultures for strain growth characterisation were previously acclimated for at least two passings to the experimental conditions (PPFD of 80 µmol s^−1^m^−2^ with a 14 h:10 h light:dark cycle, at a constant temperature of 19.0 ± 1.0 °C). A laboratory-scale growth experiment was performed in triplicate for 13 days. Each replicate of 400 mL fresh L1 medium [47] was inoculated to an initial concentration of 1.69 × 10^4^ cells mL^−1^.

Cell concentration (CC) was followed by cell count, every two days for 13 days, until the early stationary phase was reached [48]. Cell counts were performed in quadruplicate (3 mL per replicate) using an Utermöhl sedimentation chamber in an inverted microscope (Motic AE30 LED Digital, Motic, Kowloon Bay, Kowloon, Hong Kong). Samples were fixed with Lugol’s iodine solution before analysis.

Growth rates (*r*) were calculated as described by Equation (2) [49]:

(2) $$r=ln\left(N_{t}/N_{0}\right)/\left(T_{t}-T_{0}\right)$$

where *N_t_* is the total cells at the end of the curve, *N*_0_ is the total cells at the start of the curve, *T_t_* is the end point time and *T*_0_ is the initial point time.

The doubling time (*T*_2_) was calculated by applying Equation (3) [49]:

(3) $$T_{2}=0.6931/r$$

### 5.3. Determination of PST

#### 5.3.1. Harvest and Extraction

Both cultures were harvested at the end of the exponential phase, on the 13th day, by filtration, using GF/C fiberglass filters 0.7 μm (Whatman^TM^, Maidstone, UK). Three biological replicates of each strain and a blank (only L1 medium) were considered for the experiments. The biotoxins were extracted from *G. catenatum* cultures using the AOAC Official Method 2005.06 [50,51] with some adaptations. Briefly, 3 mL of acetic acid 1% (prepared from glacial acetic acid, HPLC grade ≥ 99.8%, from Carlo Erba) was added to each falcon tube containing the fiberglass filter with biomass. Each filter was crushed with a metal lancet to enhance the extraction yield. The samples were mixed using a vortex (Labbox, Barcelona, Spain), sonicated (Transsonic 660/H Elma) for 10 min, and heated for 5 min over a bath at 100 °C. After that, they were mixed again and placed in a beaker with ice for 5 min. Thereafter, samples were centrifuged at 7000 *g* for 10 min, at low temperature (10 °C), using a high-speed centrifuge 5430 R, Eppendorf. The supernatant was filtered into graduated glass tubes using PVDF filters 0.22 μm, 13 mm (Teknokroma). Afterwards, 3 mL of acetic acid 1% were added again to each falcon tube and mixed, using a vortex. The centrifugation and filtration processes were performed three times. At the end, the volume of all samples was adjusted to 10 mL with ultrapure water (Simplicity^®^ Water Purification System, Merck, Germany, 18.2 MΩ.cm). All samples were subjected to the solid phase extraction procedure shortly after extraction. The blank was subjected to the same experimental procedures as the samples.

#### 5.3.2. Solid-Phase Extraction (SPE) Procedure

The procedures for cleaning and fractioning the samples were based on the AOAC Official Method 2005.06 proposed for analysis of PST in shellfish but with some adaptations to our conditions. To clean-up, SPE-C18 cartridges (500 mg/3 mL, Finisterre, London, UK) were conditioned with 6 mL of methanol (≥99.9%, LC-MS Chromasolv^TM^), followed by 6 mL of ultrapure water. A total of 2.5 mL of each homogenized sample (or blank) was added to each cartridge. The flow was maintained at 2–3 mL/min and the effluent was collected in graduated tubes. A total of 2.0 mL of ultrapure water was added to the cartridges for washing and the resulting effluents were collected in the same tubes. Before final adjustment to 5.0 mL with ultrapure water, the pH of the samples was adjusted to ca. 6.5 with 1 M NaOH (p.a. from Chem-Lab) using a pH indicator paper.

For fractioning, SPE-COOH cartridges (500 mg/3 mL, Speed^TM^ Applied Separations, Allentown, PA, USA) were conditioned with 10 mL of 0.01 M ammonium acetate (≥98%, Pronalab^®^). Then, 2.0 mL of each sample extract from SPE-C18 was added to ion exchange cartridges and the effluents were collected in graduated tubes. A total of 4.0 mL of ultrapure water was added to each cartridge and collected in the same tubes. The tubes containing the effluent were removed and their final volume was adjusted to 6.0 mL. These correspond to the first fractions of each sample, which contain the C-toxins. Then, 4.0 mL of 0.05 M NaCl (p.a., ≥99.8%, Chem-Lab, Zedelgem, BELGIUM) was added to the same cartridges and the effluent was collected in other graduated tubes. These tubes were removed, and their final volume was adjusted to 4.0 mL; these extracts correspond to the second fractions (GTX1-6 and dcGTX2,3). Finally, 5.0 mL of 0.3 M NaCl was added to the same cartridges and the corresponding effluents (third fractions) were collected in other graduated tubes. All samples and the blank, after extraction (Section 5.3.1) and after cleaning and fractionation procedures by SPE, were stored in dark glass vials in the refrigerator until oxidation for subsequent analysis by HPLC-FLD.

#### 5.3.3. Analysis by HPLC-FLD with Pre-Column Oxidation

The samples were analysed by HPLC–FLD (Shimadzu, Prominence-i LC-2030C Plus), according to an adaptation of Lawrence’s method (AOAC Official Method 2005.06). The detailed features of this adapted method and the equipment used are described in the SI and in a previous publication [52]. All solvents were previously filtered, using PVDF membrane filters (0.22 μm, 47 mm) from Teknokroma. The injection volumes were 30 μL or 100 μL, for solutions oxidized with peroxide or periodate, respectively. Three replicates of the injection were carried out for each sample extract.

Before analysis, all samples were oxidized to allow the detection of toxins. Sample extracts from SPE-C18 were separately preoxidized with periodate and peroxide. Sample extracts from SPE-COOH were oxidized only with periodate. The periodate reagent allows the detection of both hydroxylated and non-hydroxylated toxins; whereas the peroxide reagent is specific for non-hydroxylated toxins (dcGTX2,3; C1,2; dcSTX; GTX2,3; GTX5; STX). Additionally, the blank was subjected to both oxidation procedures to evaluate possible matrix effects.

The periodate oxidant was freshly prepared each day of the analysis by mixing equal volumes of 0.03 M of periodic acid (H_5_IO_6_ ≥ 99.5%, Riedel-deHaën), 0.3 M of ammonium formate (NH_4_HCO_2_, LC-MS grade from Carlo Erba), 0.3 M of disodium hydrogen phosphate (Na_2_HPO_4_, 99.5%, Riedel-deHaën) and adjusting the pH of the mixture to 8.2 with NaOH 0.2 M, using a pH meter (Hanna). For periodate oxidation, we adopted a procedure previously reported [53]. Briefly, 100 μL of sample extract (or standard solution) was mixed with 100 μL of ultrapure water, followed by the addition of 500 μL of periodate reagent. The mixture was stirred (using a vortex) and let to react at room temperature at least 1 min. After that, 5 μL of glacial acetic acid was added, the mixture was again stirred and let to react for 10 min at room temperature.

For peroxide oxidation, 375 μL of 1 M NaOH and 37.6 μL of H_2_O_2_ 10% (*w*/*v*), obtained from H_2_O_2_ 50% (*w*/*w*) (Scharlau), were firstly mixed in an autosampler vial (a vortex was used for the mixes). Then, 150 μL of sample extract (or standard solution) was added, and the final mixture was stirred and allowed to react for 10 min at room temperature. Then, 30 μL of glacial acetic acid was added to stop the reaction.

#### 5.3.4. Toxins Identification and Quantification

To identify and quantify PST, calibration curves with 4–6 concentration levels of each toxin or mixtures of toxins were performed in aqueous solution. For that, the eleven certified reference materials (CRM) available on the market were considered: dcSTX (from National Research Council Canada, Halifax, Canada); STX; dcGTX2,3; C1,2; C3,4; GTX1,4; GTX2,3; GTX5, GTX6, NEO and dcNEO (from CIFGA laboratory S.A., Lugo, Spain). Identification of the toxins in the sample extracts was made in correspondence with the retention times (R_t_) of the CRM and their quantification through the calibration curve for the corresponding toxin.

### 5.4. Statistical Analysis

Data were calculated as the mean of three independent replicates ± standard deviation (SD). Normality and homogeneity of variance were tested using the Shapiro and Levene tests, respectively. When found, outliers were removed according to the interquartile range (IQR). An independent samples *t*-test was used to assess differences between the means of the two tested strains. Only results with *p* < 0.05 were considered statistically different. In addition, analysis of variance (ANOVA) was performed to test differences in toxin content. Statistical treatment was performed using R software (version 4.1.0) and Microsoft^®^ Excel^®^ (version 2209).

## Figures and Tables

**Table 1:** PST profile of *G. catenatum* cultures used in this study. The values correspond to the average ± SD. TEFs presented were proposed by the EFSA.

Strain		IO13-25-02		IO13-26-02	
Toxin	TEF (EFSA)	Concentration fmol Cell^−1^	Molar Fraction (%)	Concentration fmol Cell^−1^	Molar Fraction (%)
dcGTX2,3	0.4	1.0 ± 0.2	6 ± 1	0.57 ± 0.02	3.0 ± 0.1
C1,2	0.1	5.6 ± 1.2	31 ± 4	2.8 ± 0.5	15 ± 1
dcSTX	1.0	6.8 ± 1.0	40 ± 8	9.5 ± 0.2	49 ± 3
GTX5 (or B1)	0.1	2.5 ± 0.4	14 ± 2	1.8 ± 0.2	9.8 ± 0.4
GTX6 (or B2)	0.1	3.4 ± 4.7	9 ± 16	4.2 ± 0.6	23 ± 2

**Table 2:** Toxin profiles (mol%) of *G. catenatum* strains from Iberian Peninsula.

	Region, Date	Strain	dcGTX2,3	C1,2	dcSTX	GTX5	GTX2,3	STX	GTX6	C3,4	dcNEO	GTX1,4	NEO	Ref.
Portugal	Lisbon bay, 2018	IO13-25-02	6 ± 1	31 ± 4	40 ± 8	14 ± 2	nd	<LOQ	9 ± 16	<LOQ	nd	<LOQ	<LOQ	This study
	Lisbon bay, 2018	IO13-26-02	3.0 ± 0.1	15 ± 1	49 ± 3	9.8 ± 0.4	nd	<LOQ	23 ± 2	<LOQ	nd	<LOQ	<LOQ	This study
	Lisbon bay, 2007		2	43	15	22	nd	nd	8	3	7	---	---	[5] ^+^
	Lisbon bay, 2007	C37/07	3.2	34.3	4.1	23.6	---	---	16.2	17.1	1.5	---	---	[46]
	Espinho, 2005	IO13-04	3 *	67 *	3	2	5	nd	2	14 *	2	2 *	nd	[27] ^+^
	Algarve, 2003 2008													
		IO13-01	---	22.1	1.4	41.4	---	---	15.5	19.6	---	---	---	[3]
		IO13-17 ^++^	---	44.8	35.4	19.8	---	---	---	---	---	---	---	
	Lisbon bay, 2003 2005	IO13-02 IO13-06 ^++^	--- ---	13.0 41.4	2.2 35.1	23.1 23.5	--- ---	--- ---	27.4 ---	34.3 ---	--- ---	--- ---	--- ---	[3]
	Aveiro, 2010 2011	IO13-22 IO13-24	--- ---	9.0 14.6	1.5 0.9	24.0 13.0	--- ---	--- ---	30.8 39.0	34.7 32.5	--- ---	--- ---	--- ---	[3]
	Aguda, 1989	PT02	8.5	30.8	5.3	27.4	2.1	0.7	12.5	12.7	---	nd	---	[7] ^+^
	Portugal, unknown		8.1	31.8	5.5	26.9	1.8	0.6	15.1	10.4	---	---	---	[8]
Spain	Ria de Vigo, 1985	5 strains	1.0–7.7	13.5–26.3	3.3–4.6	18.5–37.1	0.3–3.3	nd	15.3–34.8	8.0–14.8	---	nd	---	[7] ^+^
	Galicia, 1985-1993	5 strains	nd–1.8 (4)	9.9–66.0 * (5)	0.3–14.7 (5)	18.4–27.1 (5)	nd–0.09 * (1)	2.0–13.0 (5)	2.9–48.4 (5)	nd–21.2 * (3)	---	nd–1.8 (2)	nd–17.4 (1)	[9] ^+++^
	Andalucia, 1999	11 strains	0.9–7.7 (11)	12.5–97.4 * (11)	nd–3.7 (9)	nd–32.5 (9)	nd–4.9 * (3)	nd	nd–37.6 (8)	nd–34.6 * (9)	---	nd	nd–57.8 (7)	[9] ^+++^
	Spain, unknown		4.5	22.7	3.5	29.2	2,0	nd	22.8	15.3	---	---	---	[8]

* Sum of epimeric pair. ^+^ Percentages recalculated by us from the authors’ data, excluding GC toxins. ^++^ Percentages recalculated by us from the authors’ data, excluding dcGTX1+2. ^+++^ Values normalized by exclusion of doSTX. (n)—number of strains in which the toxin was identified. LOQ—Limit of quantification; nd—not detected.

## Data Availability

Not applicable.

## Supplementary Materials

The following supporting information can be downloaded at: https://www.mdpi.com/article/10.3390/toxins14110762/s1, Figure S1: Toxin concentration for both strains of *G. catenatum*; Table S1: Limits of quantification (LOQ), limits of detection (LOD) (in μmol/L) and determination coefficients for each toxin (lower values); details about HPLC-FLD method.

## References

[1] Kobayashi J., Kubota T. (2010). Bioactive Metabolites from Marine Dinoflagellates. Compr. Nat. Prod. II Chem. Biol..

[2] Assunção J., Guedes A., Malcata F. (2017). Biotechnological and Pharmacological Applications of Biotoxins and Other Bioactive Molecules from Dinoflagellates. Mar. Drugs.

[3] Silva T., Caeiro M.F., Costa P.R., Amorim A. (2015). Gymnodinium Catenatum Graham Isolated from the Portuguese Coast: Toxin Content and Genetic Characterization. Harmful Algae.

[4] Liu M., Gu H., Krock B., Luo Z., Zhang Y. (2020). Toxic Dinoflagellate Blooms of Gymnodinium Catenatum and Their Cysts in Taiwan Strait and Their Relationship to Global Populations. Harmful Algae.

[5] Costa P.R., Robertson A., Quilliam M.A. (2015). Toxin Profile of Gymnodinium Catenatum (Dinophyceae) from the Portuguese Coast, as Determined by Liquid Chromatography Tandem Mass Spectrometry. Mar. Drugs.

[6] Band-Schmidt C., Bustillos-Guzmán J., Morquecho L., Gárate-Lizárraga I., Alonso-Rodríguez R., Reyes-Salinas A., Erler K., Luckas B. (2006). Variations of PSP Toxin Profiles during Different Growth Phases in Gymnodinium Catenatum (Dinophyceae) Strains Isolated from Three Locations in the Gulf of California, Mexico. J. Phycol..

[7] Negri A.P., Bolch C.J.S., Geier S., Green D.H., Park T.-G., Blackburn S.I. (2007). Widespread Presence of Hy-drophobic Paralytic Shellfish Toxins in Gymnodinium Catenatum. Harmful Algae.

[8] Negri A.P., Bolch C.J.S., Blackburn S.I., Dickman M., Llewellyn L.E., Mendez S., Hallegraeff G.M., Blackburn S.I., Bolch C.J.S., Lewis R.J. (2001). Paralytic shellfish toxins in Gymnodinium catenatum strains from six countries. Harmful Algal Blooms 2000, Proceedings of the Ninth International Conference on Harmful Algal Blooms, Hobart, Tasmania, Australia, 7–11 February 2000.

[9] Ordás M.C., Santiago F., Franco J.M., Ordás A., Figueiras A. (2004). Toxin and Molecular Analysis of Gym-nodinium Catenatum (Dinophyceae) Strains from Galicia (NW Spain) and Andalucia (S Spain). J. Plankton Res..

[10] Hallegraeff G.M., Anderson D.M., Belin C., Bottein M.Y., Bresnan E., Chinain M., Enevoldsen H., Iwataki M., Karlson B., McKenzie C.H. An Unprecedented Analysis on Global Harmful Algal Blooms Launched by IOC. https://ioc.unesco.org/news/unprecedented-analysis-global-harmful-algal-blooms-launched-ioc.

[11] Hallegraeff G.M., Anderson D.M., Belin C., Dechraoui Bottein M.-Y., Bresnan E., Chinain M., Enevoldsen H., Iwataki M., Karlson B., McKenzie C.H. (2021). Perceived Global Increase in Algal Blooms Is At-tributable to Intensified Monitoring and Emerging Bloom Impacts. Commun. Earth Environ..

[12] Lagos N.W., Andrinolo D., Botana L.M. (2000). Paralytic Shellfish Poisoning (PSP): Toxicology and Kinetics. Seafood and Freshwater Toxins: Pharmacology, Physiology, and Detection.

[13] Gessner B.D., Bell P., Doucette G.J., Moczydlowski E., Poli M.A., van Dolah F., Hall S. (1997). Hypertension and Identification of Toxin in Human Urine and Serum Following a Cluster of Mussel-Associated Para-lytic Shellfish Poisoning Outbreaks. Toxicon.

[14] García C., del Carmen Bravo M., Lagos M., Lagos N. (2004). Paralytic Shellfish Poisoning: Post-Mortem Analysis of Tissue and Body Fluid Samples from Human Victims in the Patagonia Fjords. Toxicon.

[15] Montebruno D. (1993). Paralytic Shellfish Poisoning in Chile. Med. Sci. Law.

[16] EUR-Lex-32004R0853-EN-EUR-Lex. https://eur-lex.europa.eu/legal-content/EN/TXT/?uri=CELEX%3A32004R0853.

[17] Wiese M., D’Agostino P.M., Mihali T.K., Moffitt M.C., Neilan B.A. (2010). Neurotoxic Alkaloids: Saxitoxin and Its Analogs. Mar. Drugs.

[18] Hall S., Strichartz G., Moczydlowski E., Ravindran A., Reichardt P.B., Hall S., Strichartz G. (1990). The Saxitoxins-Sources, Chemistry, and Pharmacology. Marine Toxins-Origin, Structure, and Molecular Pharmacology.

[19] Negri A., Stirling D., Quilliam M., Blackburn S., Bolch C., Burton I., Eaglesham G., Thomas K., Walter J., Willis R. (2003). Three Novel Hydroxybenzoate Saxitoxin Analogues Isolated from the Dinoflagellate Gym-nodinium Catenatum. Chem. Res. Toxicol..

[20] Leal J.F., Cristiano M.L.S. (2022). Marine Paralytic Shellfish Toxins: Chemical Properties, Mode of Action, Newer Analogues, and Structure–Toxicity Relationship. Nat. Prod. Rep..

[21] Llewellyn L., Negri A., Quilliam M. (2004). High Affinity for the Rat Brain Sodium Channel of Newly Discovered Hydroxybenzoate Saxitoxin Analogues from the Dinoflagellate Gymnodinium Catenatum. Toxicon.

[22] Durán-Riveroll L.M., Cembella A.D., Band-Schmidt C.J., Bustillos-Guzmán J.J., Correa-Basurto J. (2016). Docking Simulation of the Binding Interactions of Saxitoxin Analogs Produced by the Marine Dinoflagellate Gymnodinium Catenatum to the Voltage-Gated Sodium Channel Nav1.4. Toxins.

[23] (2020). WHO Cyanobacterial Toxins: Saxitoxins.

[24] Hallegraeff G.M., Blackburn S.I., Doblin M.A., Bolch C.J.S. (2012). Global Toxicology, Ecophysiology and Popu-lation Relationships of the Chainforming PST Dinoflagellate Gymnodinium Catenatum. Harmful Algae.

[25] Graham H.W. (1943). Gymnodinium Catenatum, a New Dinoflagellate from the Gulf of California. Trans. Am. Microsc. Soc..

[26] Carvalho I.L.d., Pelerito A., Ribeiro I., Cordeiro R., Núncio M.S., Vale P. (2019). Paralytic Shellfish Poisoning Due to Ingestion of Contaminated Mussels: A 2018 Case Report in Caparica (Portugal). Toxicon X.

[27] Costa P.R., Braga A.C., Turner A.D. (2018). Accumulation and Elimination Dynamics of the Hydroxybenzoate Saxitoxin Analogues in Mussels Mytilus Galloprovincialis Exposed to the Toxic Marine Dinoflagellate Gymnodinium Catenatum. Toxins.

[28] Franca S., Almeida J.F. (1989). Paralytic Shellfish Poisons in Bivalve Molluscs on the Portugese Coast Caused by a Bloom of the Dinoflagellate Gymnodinium Catenatum. Red Tides: Biology, Envionmental Science and Toxicology.

[29] (2009). EFSA Marine Biotoxins in Shellfis –Saxitoxin Group. EFSA J..

[30] Rodriguez-Navarro A.J.M.D., Lagos N.P.D., Lagos M.M.D., Braghetto I.M.D., Csendes A.M.D., Hamilton J.M.D., Figueroa C.M.D., Truan D.M.S., Garcia C.M.S., Rojas A.M.D. (2007). Neosaxitoxin as a Local An-esthetic: Preliminary Observations from a First Human Trial. Anesthesiol. J. Am. Soc. Anesthesiol..

[31] Adams H.J., Blair M.R., Takman B. (1976). The Local Anesthetic Activity of Saxitoxin Alone and with Vasocon-strictor and Local Anesthetic Agents. Arch. Int. Pharmacodyn. Ther..

[32] Kohane D.S., Lu N.T., Gökgöl-Kline A.C.G., Berde C.B., Shubina M., Kuang Y., Hall S., Strichartz G.R. (2000). The Local Anesthetic Properties and Toxicity of Saxitonin Homologues for Rat Sciatic Nerve Block In Vivo. Reg. Anesth. Pain Med..

[33] (2016). FAO/WHO Technical Paper on Toxicity Equivalency Factors for Marine Biotoxins Associated with Bi-Valve Molluscs.

[34] Lin Z.R., Geng H.X., Zhang Q.C., Chen Z.F., Dai L., Yu R.C. (2022). Toxin Production of Dinoflagellate Gym-nodinium Catenatum Isolated from the East China Sea. Harmful Algae.

[35] Fernández-Herrera L.J., Band-Schmidt C.J., Zenteno-Savín T., Leyva-Valencia I., Hernández-Guerrero C.J., Muñoz-Ochoa M. (2021). Cell Death and Metabolic Stress in Gymnodinium Catenatum Induced by Allelopathy. Toxins.

[36] Han K.H., Kim H.J., Li Z., Youn J.Y., Kwak K.Y., Seo M.H., Hwang J., Lee S.D., Yun S.M., Oh S.J. (2020). Effects of Different Nutrient and Trace Metal Concentrations on Growth of the Toxic Dinoflagellate Gym-nodinium Catenatum Isolated from Korean Coastal Waters. Sustainability.

[37] Band-Schmidt C.J., Bustillos-Guzmán J.J., Hernández-Sandoval F.E., Núñez-Vázquez E.J., López-Cortés D.J. (2014). Effect of Temperature on Growth and Paralytic Toxin Profiles in Isolates of Gymnodinium Catenatum (Dinophyceae) from the Pacific Coast of Mexico. Toxicon.

[38] Green D., Hart M., Blackburn S., Bolch C. (2010). Bacterial Diversity of Gymnodinium Catenatum and Its Relationship to Dinoflagellate Toxicity. Aquat. Microb. Ecol..

[39] Sako Y., Yoshida T., Uchida A., Arakawa O., Noguchi T., Ishida Y. (2001). Purification and Characterization of a Sulfotransferase Specific to N-21 of Saxitoxin and Gonyautoxin 2+3 from the Toxic Dinoflagellate Gymnodinium Catenatum (Dinophyceae). J. Phycol..

[40] Yoshida T., Sako Y., Uchida A., Kakutani T., Arakawa O., Noguchi T., Ishida Y. (2002). Purification and Characterization of Sulfotransferase Specific to O-22 of 11-Hydroxy Saxitoxin from the Toxic Dinoflagel-late Gymnodinium Catenatum (Dinophyceae). Fish Sci..

[41] Wang D., Zhang S., Hong H. (2007). A Sulfotransferase Specific to N-21 of Gonyautoxin 2/3 from Crude En-zyme Extraction of Toxic Dinoflagellate Alexandrium Tamarense CI01. Chin. J. Oceanol. Limnol..

[42] Buzy A., Thibault P., Laycock M. (1994). v Development of a Capillary Electrophoresis Method for the Charac-terization of Enzymatic Products Arising from the Carbamoylase Digestion of Paralytic Shellfish Poisoning Toxins. J. Chromatogr. A.

[43] Fast M.D., Cembella A.D., Ross N.W. (2006). In Vitro Transformation of Paralytic Shellfish Toxins in the Clams Mya Arenaria and Protothaca Staminea. Harmful Algae.

[44] Lin H.-P., Cho Y., Yashiro H., Yamada T., Oshima Y. (2004). Purification and Characterization of Paralytic Shellfish Toxin Transforming Enzyme from Mactra Chinensis. Toxicon.

[45] Oshima Y., Blackburn S.I., Hallegraeff G.M. (1993). Comparative Study on Paralytic Shellfish Toxin Profiles of the Dinoflagellate Gymnodinium Catenatum from Three Different Countries. Mar. Biol..

[46] Costa P.R., Pereira P., Guilherme S., Barata M., Nicolau L., Santos M.A., Pacheco M., Pousão-Ferreira P. (2012). Bio-transformation Modulation and Genotoxicity in White Seabream upon Exposure to Paralytic Shellfish Toxins Produced by Gymnodinium Catenatum. Aquat. Toxicol..

[47] Guillard R.R.L., Hargraves P.E. (1993). Stichochrysis Immobilis Is a Diatom, Not a Chrysophyte. Phycologia.

[48] Singh P., Gupta S.K., Guldhe A., Rawat I., Bux F. (2015). Microalgae Isolation and Basic Culturing Techniques. Handbook of Marine Microalgae: Biotechnology Advances.

[49] Wood A.M., Everroad R.C., Wingard L.M. (2005). Measuring Growth Rates in Microalgal Cultures. Algal Culturing Techniques.

[50] Horwitz W., Latimer G.W. (2005). Unknown AOAC, Paralytic Shellfish Poisoning Toxins in Shellfish. Prechromatographic and Liquid Chromatography with Fluorescence Detection. First Action, Official Method 2005.06. AOAC Official Methods of Analysis.

[51] Lawrence J.F., Niedzwiadek B., Menard C. (2005). Quantitative Determination of Paralytic Shellfish Poisoning Toxins in Shellfish Using Prechromatographic Oxidation and Liquid Chromatography with Fluorescence Detection: Collaborative Study. J. AOAC Int..

[52] Leal J.F., Cristiano M.L.S. (2022). Revisiting the HPLC-FLD Method to Quantify Paralytic Shellfish Toxins: C3,4 Quantification and the First Steps towards Validation. Toxins.

[53] Rodríguez P., Alfonso A., Botana A.M., Vieytes M.R., Botana L.M. (2010). Comparative Analysis of Pre- and Post-Column Oxidation Methods for Detection of Paralytic Shellfish Toxins. Toxicon.

