# Supplementary material for: Toxin Profile of Two Gymnodinium catenatum Strains from Iberian Coastal Waters
Source: Toxins (Basel). 2022 Nov 4;14(11):762. doi: 10.3390/toxins14110762 (PMC9699220; doi:10.3390/toxins14110762)
Supplement: Supplementary file 1 [file toxins-14-00762-s001.zip › toxins-2002392 SM .pdf]

# Supplementary Materials: **Toxin profile of two *Gymnodinium catenatum* strains from Iberian coastal waters**

**Joana F. Leal, Gabriel Bombo, Hugo Pereira, Bernardo Vicente, Ana Amorim and Maria L. S. Cristiano**

**Table S1.** Limits of quantification (LOQ), limits of detection (LOD) (in μmol/L) and determination coefficients for each toxin (lower values). Calibration curves performed between 0.04 and 3 μM.

| Toxin | LOD | LOQ | *R2* |
|---|---|---|---|
| dcGTX2,3 | 0,01 | 0,02 | 0.9998 |
| C1,2 | 0,01 | 0,04 | 0.9999 |
| dcSTX | 0,02 | 0,07 | 0.9995 |
| GTX2,3 | 0,01 | 0,05 | 0.9996 |
| GTX5 (or B1) | 0,01 | 0,02 | 0.9999 |
| STX | 0,09 | 0,30 | 0.9994 |
| C3,4 | 0,03 | 0,12 | 0.9994 |
| GTX1,4 | 0,08 | 0,27 | 0.9990 |
| dcNEO | 0,13 | 0,42 | 0.9994 |
| GTX6 (or B2) | 0,02 | 0,07 | 0.9992 |
| NEO | 0,05 | 0,16 | 0.9993 |

LOD and LOQ were determined as (3σ)/m and (10σ)/m, where σ is the residual standard deviation of the regression line and m is the slope of the calibration curve (ICH, 2005; Miller & Miller, 2005).

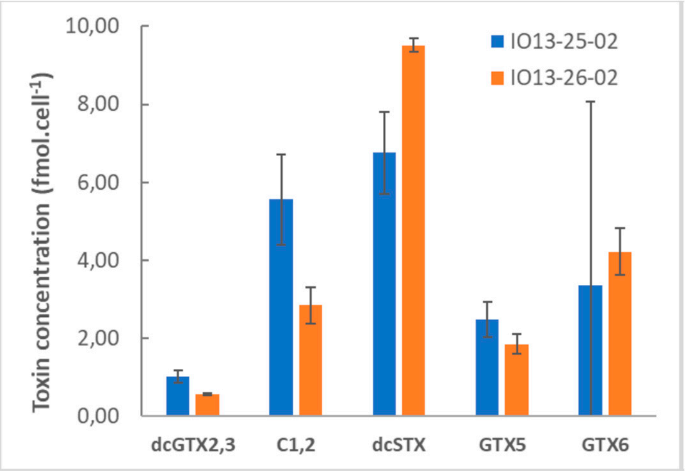


**Figure S1** – Toxin concentration for both strains of G. catenatum. Hla: alpha-hemolysin encoding gene.

## HPLC-FLD analysis:

HPLC-FLD is composed by a quaternary pump, a refrigerator autosampler, a column oven and a spectrofluorometric detector (RF-20A XS). The temperatures used were 10 °C, 25 °C and 30 °C in the autosampler, column oven and cell detector, respectively. A reversed-phase C18 column (Mediterranea Sea18, Teknokroma), 25 cm x 0.46 cm (5 µm), and an ultraguardTM column (Sea18 10 x 3.2 mm, Teknokroma) were used. Ammonium formate 0.1 M (adjusted to pH 6 with acetic acid 0.1 M) and acetonitrile (HPLC grade, Honeywell) were applied as mobile phase A and B, correspondingly. The gradient elution conditions were as follow. First 6 min: 1-5 % phase B; between 6 and 13 min: 5-28 % phase B; between 13 and 16 min: 28-1% phase B; between 16 and 19 min: 1% phase B. The flow rate was 1.5 mL/min. Fluorescence detection was achieved at 340 nm (excitation) and 395 nm (emission).
